# Supplementary figures and images for: Association between Dietary Cholesterol and Their Food Sources and Risk for Hypercholesterolemia: The 2012–2016 Korea National Health and Nutrition Examination Survey
Source: Nutrients. 2019 Apr 15;11(4):846. doi: 10.3390/nu11040846 (PMC6520795; doi:10.3390/nu11040846)

### Supplementary Material:

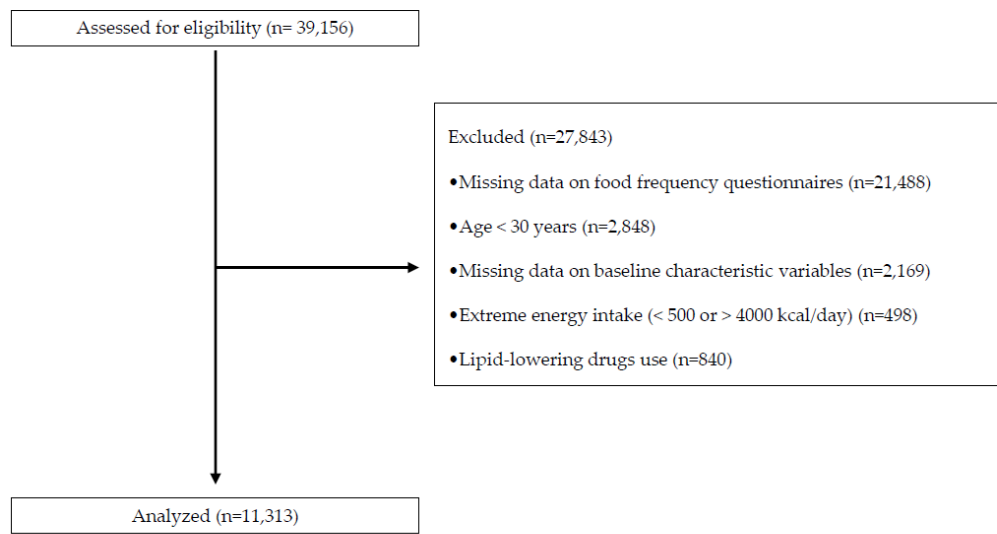

**Figure S1.** Flowchart of participants' inclusion and exclusion.

Supplement: Supplementary file 1 [file nutrients-11-00846-s001.pdf]
